# Suggesting global insights to local challenges: expanding financing of rehabilitation services in low and middle-income countries

**DOI:** 10.3389/fresc.2024.1305033

**Published:** 2024-04-22

**Authors:** Abdulgafoor M. Bachani, Jacob A. Bentley, Hunied Kautsar, Rachel Neill, Antonio J. Trujillo

**Affiliations:** Johns Hopkins International Injury Research Unit, Health Systems Program, Department of International Health, Johns Hopkins University Bloomberg School of Public Health, Baltimore, MD, United States

**Keywords:** rehabilitation, health financing, review, health economics, medical cost

## Abstract

**Purpose:**

Following the rapid transition to non-communicable diseases, increases in injury, and subsequent disability, the world—especially low and middle-income countries (LMICs)—remains ill-equipped for increased demand for rehabilitative services and assistive technology. This scoping review explores rehabilitation financing models used throughout the world and identifies “state of the art” rehabilitation financing strategies to identify opportunities and challenges to expand financing of rehabilitation.

**Material and methods:**

We searched peer-reviewed and grey literature for articles containing information on rehabilitation financing in both LMICs and high-income countries.

**Results:**

Forty-two articles were included, highlighting various rehabilitation financing mechanism which involves user fees and other innovative payment as bundled or pooled schemes. Few studies explore policy options to increase investment in the supply of services.

**Conclusion:**

this paper highlights opportunities to expand rehabilitation services, namely through promotion of private investment, improvement in provider reimbursement mechanism as well as expanding educational grants to bolster labor supply incentive, and the investment in public and private insurance schemes. Mechanisms of reimbursement are frequently based on global budget and salary which are helpful to control cost escalation but represent important barriers to expand supply and quality of services.

## Implications for rehabilitation

• New financing models for rehabilitation need to balance both supply and demand aspects of rehabilitation services.
• When designing new mechanisms to fund rehabilitation services in LMICs, stakeholders should weigh in the inclusion of long-term services, prevent adverse selection by public and private insurance, and develop an optimal insurance package that is context-specific yet cost-effective.
• While the concept of an optimal insurance package that is both context-specific and cost-effective is pivotal in the realm of rehabilitation services, current literature provides limited specific examples of such models. This gap highlights a significant challenge for community-based rehabilitation professionals: balancing the diverse rehabilitation needs of various communities with the economic constraints of insurance packages. This area presents a fertile ground for future research.
• There is ample room to combine financial incentives and non-financial rewards to expand labor supply and training for rehabilitation services. For instance, offering professional development opportunities, such as specialized training in emerging rehabilitation techniques or certifications, can be a significant motivator for healthcare professionals. Another example is the recognition of excellence in rehabilitation care through awards or public acknowledgment, which can boost morale and encourage a commitment to high-quality service provision.
• More research is needed to explore various rehabilitation services and assistive technology financing strategy in low and middle-income countries.

## Introduction

Epidemiologic transition from communicable to non-communicable diseases is rapidly increasing the global burden of disabilities. This includes rapid urbanization, increased motorization increasing the risk of injuries, and a rapidly growing older adults population living with long-term diseases and disabilities. The world—especially low and middle-income countries (LMICs)—remains ill-equipped to cope with the constant increase in demand for rehabilitative services and assistive technology (AT) (1, 2). Under this changing environment, a fundamental challenge for countries is how to efficiently channel domestic and international funds to cover increasing demands for rehabilitation services.

In many countries, financing of rehabilitation services has been poorly integrated into national health financing schemes, which has resulted in unmet needs, and disconnect between what is needed by the population and what is financed and made available. Unmet needs are not only the consequence of increasing needs, demand, and lack of availability of services, but also inadequate funding and lack of economic incentives to expand both private and public investment in the supply of services (2, 3). New financial models need to balance both demand and supply aspects to integrate rehabilitations services into national financing schemes.

Additionally, there are many misconceptions related to direct and indirect costs associated with rehabilitation services and AT, which are a significant obstacle to developing and expanding this sector (3, 4). The fundamental role of human resources in the cost of production of services, cost escalation over time, and productivity of the sector are not quite understood and missed in the planning of financing policies to promote the growth of the sector (5, 6). Obstacles to integrate the private sector and remove barriers to promote fair competition and new entry of providers are elements usually ignored in the policy debates (6).

As is common in other services, uneven geographic distribution of services within a country and gaps in quality of services between private and public providers also contribute to limiting access, and not only have negative impacts on the health and well-being of individual patients but also on the quality of life and general well-being of their families as well as the larger society (7–9). These implications go beyond health, trapping individuals and their families in poverty due to lack of educational and work opportunities and social isolation due to stigma and discrimination (8).

To date, there has been a dearth of information that highlights the various financing mechanisms for rehabilitation services around the world, as well as effective and efficient rehabilitation financing schemes that can serve as positive examples which could be adapted to the LMIC setting. Synthesizing such information will help to identify “state-of-the-art” rehabilitation financing strategies and possible interventions to achieve such strategies. We include as financing mechanisms, the instruments used to raise funds to cover the needs of the system, as well as the rules in place to allocate the funds among providers.

This paper presents a scoping review that, while recognizing the historic 2023 World Health Assembly resolution on strengthening rehabilitation in health systems, aims to specifically understand the financial aspects of managing disability and developing rehabilitation infrastructure in LMICs. To ensure a focused inquiry, our review concentrates on four key objectives:

1. Defining and describing specific models of rehabilitation financing, including private and public insurance, state, public-private, and NGO mechanisms, to raise funds for the sector.
2. Identifying precise mechanisms used by public and private payers to allocate funds among both private and public providers, focusing on the financial dynamics.
3. Describing detailed payment mechanisms to reimburse physicians and service providers, concentrating on the financial transactions.
4. Identifying finance-related challenges for the workforce, emphasizing on productivity improvement and training in new technologies, which affect the coordination of rehabilitation services in LMICs and the establishment of sustainable links between hospital-based and other programs.

This focused approach allows us to delve deeply into the financial dimensions of rehabilitation services in LMICs, providing a clear and concise understanding of the challenges and opportunities within this specific scope.

## Materials and methods

This scoping review was conducted according to PRISMA (Preferred Reporting Items for Systematic Reviews and Meta-Analyses) and PRISMA extension for scoping reviews (PRISMA-ScR) guidelines (10, 11). This review was guided by an adapted PICOS (Population, Intervention, Comparison/Context, Outcome, Setting) framework, which effectively encompasses the Population, Concept, and Context guide recommended for scoping reviews. Our extended framework includes five components: P (Population) to define the group of interest, I (Intervention) to describe the rehabilitation financing models being assessed, C (Comparison/Context) to consider the various settings and circumstances within LMICs, O (Outcome) to identify the specific financial outcomes of interest, and S (Setting) to detail the specific environments where rehabilitation services are provided. This approach ensures a thorough and targeted search strategy, aligning with best practices for scoping reviews (Table 1).

**Table 1:** PICOS questions.

(1) Should community delivered rehabilitation services compared to hospital/clinic or facility-based rehabilitation be offered to people experiencing physical or mental disabilities?
(2) Should reallocation/ redistribution of financial resources compared traditional funding mechanisms be used to assist people with disability?
(3) Should rehabilitation services that have user fees compared to rehabilitation services that do not have user fees be offered to people experiencing physical or mental disabilities?
(4) Should privately funded rehabilitation services compared to publicly funded rehabilitation services be offered to people experiencing physical or mental disabilities?
(5) Should rehabilitation services that provide free care or subsidized care for the poor compared to rehabilitation services that do not provide free care or subsidized care for the poor be offered to people experiencing physical or mental disabilities?
(6) Should health insurance cover rehabilitation services compared to health insurance does not cover rehabilitation services be offered to people experiencing physical or mental disabilities?
(7) Should alternative methods of reimbursement use to increase the productivity of the rehabilitation sector?
(8) What are the market and institutional barriers to expand the supply of rehabilitation services?

The target population, which is consistent across the eight PICOS questions, includes all individuals who experience physical and/or mental disabilities. Following the International Classification of Functioning (ICF) model, “disability” is inclusively conceptualized for the purposes of this project to fully capture the financial implications of the services provided to individuals with long-term health conditions and disease as well as those who have sustained injuries (12). Financing strategies for rehabilitation services were selected based on background research, and recommendations in the World Report on Disability (13). These include relocation or redistribution of existing resources, user fees, a combination of public and private financing, alternative methods of payment, increase access to disability interventions among those with limited resources, and expansion of health insurance coverage.

Our target outcomes have been defined to align with the specific aims of our review, focusing on the financial aspects of rehabilitation services for individuals with disabilities and the operational efficiency of rehabilitation facilities. For individuals with disabilities, we narrowed our inquiry to specifically investigate the financial barriers they encounter in accessing rehabilitation services. This includes an analysis of the direct and indirect costs of care, as well as the economic impact on their families, who often assume caregiving responsibilities. Our aim is to elucidate the financial challenges and potential solutions that can improve access to rehabilitation services for this population.

Regarding rehabilitation facilities, we have focused our examination on their financial efficiency and effectiveness. This entails assessing their resource allocation strategies, funding mechanisms, and the cost-effectiveness of service delivery models. Our objective is to identify best practices and potential areas for improvement in the financing and management of rehabilitation facilities.

Lastly, in the supply component, we explore alternative methods of payment to reimburse and increase the productivity of public and private providers of rehabilitation of services. As a last item of the supply component, we also study current barriers to expand the supply of both public and private rehabilitation services.

Through this refined focus, we aim to provide a clearer and more targeted analysis of the financial dimensions of rehabilitation services, both from the perspective of individuals with disabilities and the operational lens of service providers. This approach aligns with the International Classification of Functioning (ICF) model, ensuring that our review comprehensively addresses the financial implications of disability at both the individual and systemic levels.

### Search strategy

We searched 13 bibliographic databases and grey literature sources including PubMed/Medline, Embase, the Cochrane Library, the System for Information on Grey Literature in Europe (SIGLE), Eldis, Scopus, Pro Quest Digital dissertations, OAister, Global Health, Global Health Ovid, LILACS, EconLit, PsycINFO including PsycARTICLES, PsycEXTRA, PsycCRITIQUES, and PsycTESTS for articles containing information on rehabilitation financing in both LMICs and high-income countries published up to 2021. We also enquired expert opinions on relevant scholarships relevant to our search concept and complemented it with additional hand-searching.

Based on the eight PICOS questions highlighted above, 14 concepts using a combination of controlled vocabulary (as appropriate to each database) and keyword terms were created to conduct the literature search of each PICOS question. Identified concepts include: (1) Rehabilitation, (2) Community-based/Hospital-based/Clinic-based, (3) Economics/Finance, (4) Fees, (5) Public/Private funding, (6) Insurance, (7) Integration services, (8) Resource reallocation/redistribution, (9) Equity/Pro-poor, (10) Mentally/Physically disabled persons, (11) LMICs as based on Cochrane Group and World Bank Definitions, (12) Clinical trials. Search, (13) Performance-based financing and (14) anti-competitive behaviors in rehabilitation services were adapted for each of the databases.

Based on our search results, we collapsed the 14 concepts created into six categories: *Costs of community vs. hospital/clinic-based rehabilitation services; Alternative methods for reimbursement and reallocation of financial resources; User fees; Private vs. Public funding and health insurance; Subsidized care; and Market and institutional barriers to expand the supply of rehabilitation services. We discussed each of this category in the result section*.

### Study selection

We included literature highlighting financing models for rehabilitation, the potential barriers, and contextual factors that influence the implementation of rehabilitation programs. We included rehabilitation programs for both physical and mental health conditions as well as studies that employed economic evaluation approaches. We did not limit the review to studies in LMICs because literature on financing models used/applied in HICs could help shed light on what models or aspects of these models could apply to LMICs. Because we wanted to capture all relevant studies, we did not exclude them by study type. However, during this phase, studies were rigorously assessed for their methodological rigor and relevance to our research objectives. Specifically, we implemented a stringent criterion where any study that lacked a clear and complete description of its study design or methodology was deemed insufficient for inclusion in our analysis. This criterion was crucial to ensure the reliability and validity of our review.

Our exclusion criteria were the following: commentaries, narrative and news articles, studies that focus on ethical and legal issues of rehabilitation services provision, rehabilitation services for criminals/offenders in the justice systems, rehabilitation of substance abuse or any type of addiction, psychosocial rehabilitation for primary psychiatric conditions (e.g., schizophrenia), clinical, epidemiological, pathological studies on the effectiveness of rehabilitation treatment, and studies published in languages other than English.

### Analysis

Citations identified through the search strategy were screened for duplicates. Next, we screened titles and abstracts to remove those that were clearly not relevant to the topic. Subsequently, full-text screening was done to determine citations to be included for data extraction. We extracted data using standardized forms developed specifically for this review, gathering information on citation, country, study design, setting, sample size, and key quantitative or qualitative results. The processes of title and abstract screening, full-text screening, and data extraction were done by two reviewers working independently, with discrepancies resolved by a senior study team member.

## Results

**Figure 1:**
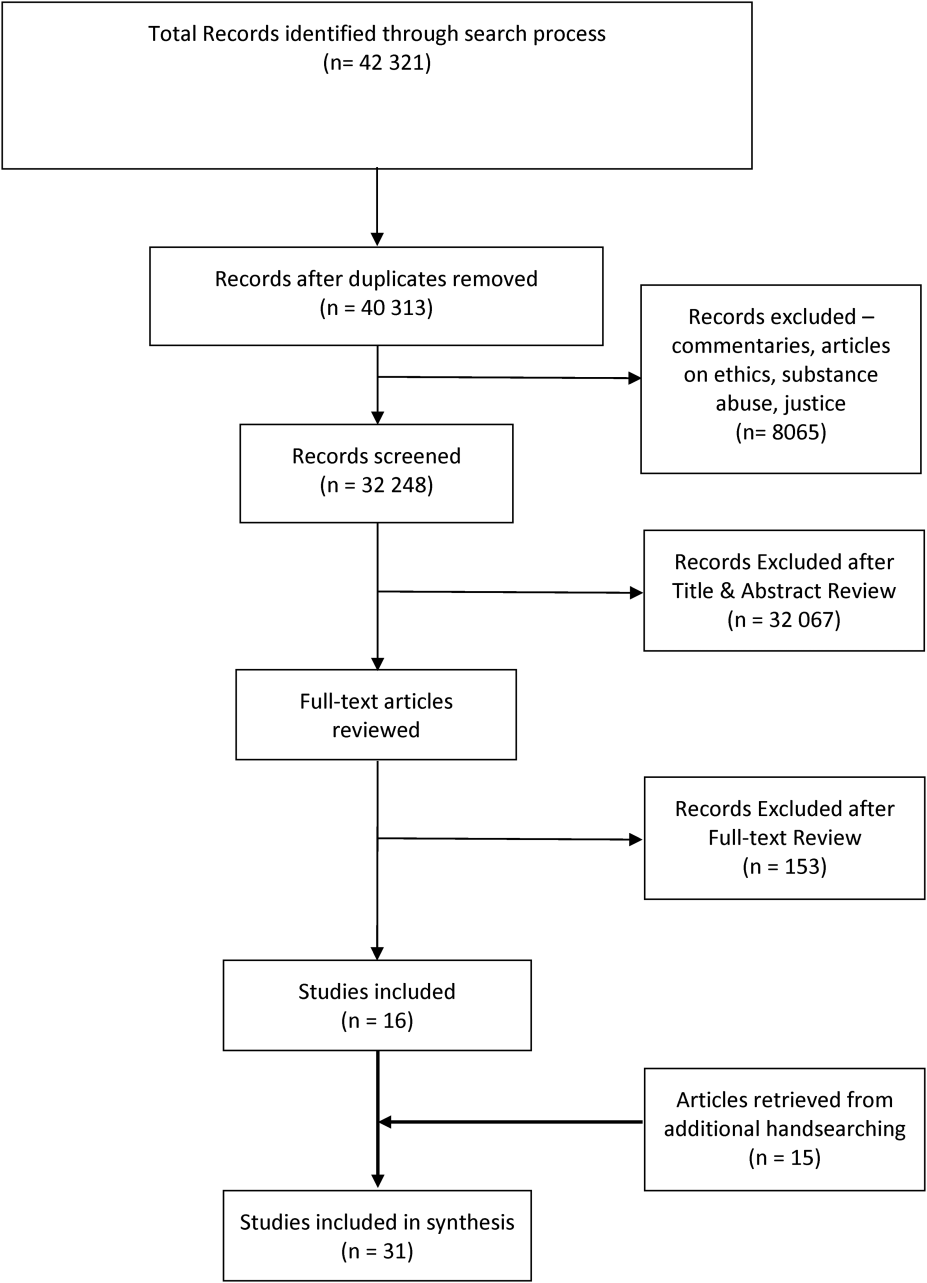
PRISMA flow diagram. exclusion criteria were the following: commentaries, narrative and news articles, studies that focus on ethical and legal issues of rehabilitation services provision, rehabilitation services for criminals/offenders in the justice systems, rehabilitation of substance abuse or any type of addiction, psychosocial rehabilitation for primary psychiatric conditions (e.g., schizophrenia), clinical, epidemiological, pathological studies on the effectiveness of rehabilitation treatment, and studies published in languages other than English.

Our database search identified 42, 321 potential articles (Figure 1). After removing duplicates, we screened the titles/abstracts of 32, 248 articles and reviewed the full text of 181 articles using our inclusion criteria. Sixteen articles met our inclusion criteria and underwent data extraction. During an additional handsearching process, we retrieved 15 additional citations. Ultimately, 42 articles were included in the synthesis. List of included studies and its characteristics are shown in Table 2.

**Table 2:** Characteristics of included studies.

Author	Year	Country	Study design/type of publication
Anderson et al. (14)	2000	Australia	RCT
Jolly et al. (15)	2007	England	RCT
McCrone et al. (16)	2009	United Kingdom	RCT
McCrone et al. (17)	1994	United Kingdom	RCT, non-blinded
Parker et al. (18)	2009	England	Pragmatic RCT
Kosycarz et al. (19)	2018	Poland	Narrative review
WHO (20)	2004	Global	Discussion paper
Sjobbema et al. (21)	2018	Netherlands	Qualitative
Gharibi et al. (22)	2021	Iran	Cross-sectional
Moghei et al. (23)	2019	Global	Cross-sectional
Obembe et al. (24)	2018	Canada	Secondary data analysis
Zaresani et al. (25)	2021	Canada	Secondary data analysis
Monkerud et al. (26)	2016	Norway	Secondary data analysis
Huckfeldt et al. (27)	2013	United States	Secondary data analysis
Brock et al. (28)	2007	Australia	Prospective cohort
Cheadle et al. (29)	1999	United States	Secondary data analysis
Dobrez et al. (30)	2010	United States	Secondary data analysis on longitudinal data & cross sectional
Dobrez et al. (31)	2004	United States	Observational study—retrospective study
Kyes et al. (32)	1999	United States	Observational study—matched control-group design
Turner-Stokes et al. (33)	2012	United States, Australia	Narrative review
Zaresani et al. (34)	2018	Canada	Secondary data analysis
Zaresani et al. (35)	2020	Canada	Secondary data analysis
Hultberg et al. (36)	2007	Sweden	Cohort
Hultberg et al. (37)	2006	Sweden	Cohort
Hultberg et al. (38)	2005	Sweden	Cohort
Hultberg et al. (39)	2003	Sweden	Qualitative
Nahar et al. (40)	2012	Bangladesh	Mixed-methods
Shields et al. (41)	2018	Global	Systematic review
Lehmann et al. (42)	2008	Global	Narrative review
Clarke et al. (43)	2021	Global	Narrative review
Naicker et al. (44)	2019	Global	Systematic review

### Costs of community vs. hospital/clinic-based rehabilitation services

Five articles reported findings on comparison between community-based and hospital/clinic-based rehabilitation services (14–18). Across the five studies, there is a high degree of heterogeneity in the study outcome of efficiency, which was assessed by mean cost per patient. One RCT (Randomized Clinical Trial) conducted in Australia (14) compared early discharge followed by home-based rehabilitation service of acute stroke patients who required rehabilitation to usual care in hospital found that the mean cost per patient was lower for patients in the home-based rehabilitation group ($8,040) compared with those who received conventional in-hospital care (US$10,054) (*p* = 0.14). On the contrary, one RCT from England (15) found that the average direct rehabilitation cost to the health service providers was significantly higher for the home-based rehabilitation program (198 GBP) compared to the hospital-based program (157 GBP) (*p* < 0.05). Additionally, two other RCTs from the United Kingdom (16, 18) found no statistically significant difference in the mean cost per patient.

### Alternative methods for reimbursement and reallocation of financial resources

The review of papers suggests that in most countries revenues to the sector comes from general taxes (19, 20). Funds are centralized in the ministry of finance (MOF) and transfer as global funds to the ministry of health. A low proportion of funds are raised using taxes at the state or local levels (20). Usually, funds allocated to rehabilitation services are embedded with a global budget to each hospital. The total of services allocated to rehabilitation services (including mental health) do not comprise more than 2% of the total hospital budget. Funding coming from private insurers pay on premium are a low proportion of the total expenses in rehabilitation.

Inclusion in the social insurance system of a separate component for disability needs usually funded through labor taxes is another mechanism to fund the system; yet it is not very prevalent among LMICs. However, the most prevalent mechanisms LMICs is through general taxes centrally collected by MOF. Literature in high income countries suggests that fixed and high disability insurance benefits may negatively impact labor force participation and the speed of health recovery (21).

Physicians and more broadly human resources for rehabilitation services are paid mostly through salary and fixed formulas which provide low incentives to increase productivity. Reimbursement methods by private insurers are based on cost of services and cap to a certain level. Bundle payments that included medical devises for rehabilitation have been used to control total medical expenditures and foster production efficiency. Few papers show impact of bundle payment on health outcomes in the field of rehabilitation.

Lastly, out-of-pocket expenditures are an important component of the funds channeled to the sector (22–25).

An important question in the scholarly work is whether to centralize or decentralized funding of rehabilitation services. Some local authorities may decide to channel local funds to cover rehabilitation services in public facilities while other may rely on central global budget. Co-financing regimes may create incentives to boost specialized rehabilitation services at the cost of primary care prevention. On the other hand, local funding may create incentives to local authorities to monitor providers’ performance more closely. In addition, cuts in local fundings for specialized rehabilitation services in public hospitals may be compensated by an increase in the supply of services by the private sector. In short, the net benefits of using co-financing public mechanisms will depend of several factors that must be carefully weighted by policy makers (26).

An analysis of Medicare (*Medicare is a federal health insurance program in the United States primarily for people aged 65 and older, but also available to younger individuals with certain disabilities or medical conditions. Established in the 60s, Medicare provides a broad range of health care services, including hospitalization, physician services, and prescription drug coverage*) provider data from 1991 through 2010 (27) showed that payment reform affects market entry and exit, which in turn may affect market structure, access to care, quality and cost of care, and patient outcomes. Payment reforms reducing average and marginal payments reduced entries and increased exits from the market. Entry effects were larger and more persistent than exit effects.

### User fees

Across six studies (28–33), two main purchasing models for rehabilitation services were identified: modified case-mix models (28, 30, 31), and a modified fee-for-service (FFS) model (29, 32). One prospective cohort study in Australia (28) explored the Case-mix and Rehabilitation Funding Tree Case-mix-based funding model (CRAFT). Payment is based on the average length of stay for the class, with short-stay cases having a higher per diem rate and longer stay cases having a lower per diem rate. This study found a significant difference between the pre-implementation year of the CRAFT system and the implementation year (z = 3.23, *P* = .001) with longer length-of-stay (LOS) in the pre-implementation stage. Functional Independence Measure (FIM) motor scores, an instrument used to measure improvement during rehabilitation at discharge, showed a significant reduction in improvement between the pre-implementation year and the implementation year. Two studies from the United States reported another model rooted from the case-mix purchasing model called the Prospective Payment System (PPS) (30, 31). The PPS model, which Medicare in the US uses, allows the reimbursement of acute inpatient care facilities to be based on the expected cost of care. Payments were based on the group classification by clinical characteristics and anticipated resource needs. The initial classification was made into a case-mix group, defined by a patient's impairment (e.g., stroke), functional level as measured by the FIM instrument, and age. Further adjustments were made on the individual patient and institutional levels. Patient-level adjustments include increased payment for comorbidities (patients with selected comorbidities are placed in 1 of 3 tiers for increased reimbursement) and high cost. Institutional adjustments include consideration of urban and rural settings and increased payment for disproportionate shares of Medicaid care (30, 31). In 2004, Dobrez et al. found that PPS reimbursements were $10,825 (37%) lower than costs. No matter how much therapy was reduced, the costs were still greater than the mean PPS reimbursement. A reduction in length of stay by 9.6 days was required to bring costs in line with the PPS reimbursement, reducing discharge cognitive function by 1.1 points (*P* < .01). The use of group therapy brought costs close to the PPS reimbursement amount and improved discharge cognitive function by 0.5 points (*P* < .10).

Two studies from the US investigated the experience of workers receiving care for occupational injuries and diseases through the Washington State Worker's Compensation Managed Care Pilot (MCP) model compared to the traditional FFS purchasing model (29, 32). The Managed care plan introduced two changes from the conventional FFS systems used by injured workers in Washington state: (1) experience-rated capitation, whereby the participating plans assumed financial risk for the services provided by agreeing to accept a prepaid amount for covered workers. Under experience-rated capitation, the capitated payment was established according to a formula based on four factors: (1) hourly rate modified for risk classification; (2) experience factor; and (3) hours worked; and (4) a primary occupational medicine delivery network, wherein the worker may choose to see any willing, authorized attending doctor to an occupational-medicine model, with care provided by a limited network of physicians trained in occupational medicine. The studies reported that the mean unadjusted medical cost per injury ($587) for the managed care group was 21.5% lower (*p* = 0.06) than for the FFS group ($748). In addition, disability costs, mainly percent on time loss and time-loss cost per injury, were significantly lower (*p* < 0.01) in the managed care group. In terms of utilization of the services, Cheadle et al. reported that there were no statistically significant differences in inpatient care between the FFS and managed care models. Furthermore, Managed care workers spent 55.8 days per 1,000 injuries in the hospital compared with 24.2 per 1,000 under FFS. There were 22% fewer outpatient visits per injury in the managed care group. Kyes et al. found that the level of satisfaction among managed-care patients was lowest regarding overall access to care. Six weeks after injury, 32% of the managed-care patients were satisfied with overall access to care, while 43% of the fee-for-service (FFS) patients were satisfied with their overall access to care (*p* < 0.001).

Few studies suggest that investment in rehabilitation services increase return to work by 10%–15% (fewer days to recover) (25, 34). Future poverty and use of social safety net services is also reduced. Lastly, studies indicate that rehabilitations services increase worker productivity by 7%–10% points (35).

### Private vs. public funding and health insurance

No studies that compared public-funded or private-funded rehabilitation services were found from our search. However, four articles reported a form of public-public partnership in financing rehabilitation in Sweden (36–39). All four articles reported the DELTA project—a collaborative effort between primary health care, social insurance, and social service. Co-financed by these three entities, the intervention health care centers ordinarily had physicians, nurses, and secretaries employed. Through co-financing by social services and social insurance, they had the opportunity to extend and intensify the rehabilitation work with other professions, such as occupational therapist, physiotherapist, social worker, and social insurance officer. For patients this implied a possibility to meet a multi-professional team located at the health center. In the 2007 article, Hultberg et al. reported that the total health care cost for an average patient in the intervention group was 1,979 Euro and 1,286 Euro (*p* = 0.007) for controls (Health centers that are not part of the DELTA project). The study also concluded that the co-financing model improved the interdisciplinary collaboration in the intervention health care centers compared to the controls.

### Subsidized care

No studies that explored free or subsidized care for rehabilitation services for the poor were found from our search. However, one mixed-methods study from Bangladesh (40) described the types of financial coping strategies used by spinal cord injury (SCI) patients receiving services from the Centre for the Rehabilitation of the Paralyzed in Bangladesh. The study found that many persons with SCI coped with financial stresses caused by rehabilitation by using savings (42.5%), mortgaging assets (12.5%), selling assets (45%), receiving loans (37.5%), begging for money (42.5%), and receiving donations from relatives (47.5%) or the community (30%). A majority (85%) of those interviewed wanted to receive financial aid in the form of interest-free loans. Over half of the participants (55.88%) wanted to have vocational training, and the rest wanted vocational training for their family members to enable them to repay the loan (40).

### Market and institutional barriers to expand the supply of rehabilitation services

Human cost of rehabilitation services is an important component of total cost of services (around 60%) (41). Thus, any policy to control cost requires reduction of salary which are difficult to implement. The flip side of this is that cost in most countries follow inflation rate as salary to the sector are negotiated according to inflation. Lack of financial incentives to increase productivity are a key problem in most LMICs (42). Increasing quality and access to rehabilitation services will require the use of both financial and other motivational policies to attract human resources to the sector. The jury is still out there about the most efficient ways to motivate human resources in the sector. The supply of services is difficult to expand as there are several regulatory barriers to expand the inflow of resources with technical education (43). LMICs should explore alternative mechanism to train the supply at lower cost. Finally, an important policy to increase supply of human resources is to expand the availability of education grants and training grants in the field of rehabilitation (44). These grants may be tailored to expand the supply of educational degrees in the field of rehabilitation. An additional option is to offer educational grants to reduce the cost of education to those individuals to get in the field of rehabilitation.

## Discussion

This international review on financing models for rehabilitation underscores that published literature documenting different rehabilitation financing models is still very limited. Across 42 articles, we found that most published literature highlights various schemes of user fees for rehabilitation services. Mainly coming from HICs, these financing schemes included modified case-mix and FFS purchasing mechanisms for rehabilitative services (28–33). Two purchasing models, namely the CRAFT model from Australia and the PPS model used by Medicare in the US, showed diverging results in terms of cost-effectiveness and efficiency (28, 30, 31). A modified FFS purchasing model called the managed care plan from the US showed a lower mean unadjusted medical cost per injury compared to the traditional FFS model (29, 32).

Our review highlights instances where rehabilitation services are co-financed by NGOs and health-service consumers. This model, observed in several LMICs, combines resources from non-governmental organizations and direct contributions from service users. It showcases a collaborative approach to funding, which can be particularly effective in settings with limited public healthcare financing.

We also discuss the complex factors influencing user fees in rehabilitation services. These include patient-level characteristics (such as income and insurance status), institutional factors (like the type of services provided and funding sources), contextual elements (such as geographic and socio-economic conditions), and caregiver status. Understanding these factors is crucial for designing equitable and sustainable user fee systems.

Our findings suggest that the financial strategies employed can influence the choice of rehabilitation services. This relationship underscores the importance of financial planning in healthcare policy, as it directly affects accessibility and the type of care patients opt for or can afford.

Thailand’s Universal Coverage Insurance Scheme provides an instructive example of integrating rehabilitation services into a national health insurance program. It offers comprehensive coverage, including rehabilitation, with a focus on equity and accessibility. The scheme is funded through general taxation, which makes it feasible for LMICs with similar economic structures. The UCS model demonstrates how a well-structured financing mechanism can enhance access to rehabilitation services, even in resource-limited settings.

Second, the Brazil's Community-Based Rehabilitation program showcases an effective way of decentralizing rehabilitation services to reach rural and underserved populations. Funded partly by government allocations and partly through public-private partnerships, the program emphasizes local community involvement and multi-sectoral collaboration. This model illustrates how LMICs can leverage local resources and partnerships to expand the reach and effectiveness of rehabilitation services.

However, satisfaction regarding overall treatment and access to care among patients in the managed care plan and FFS was relatively low. This notable finding around user fees for rehabilitation services showed that LMICs are at a more significant disadvantage: It has been well documented that user fees and out-of-pocket payments negatively impact the utilization of health services and health outcomes of the population and further decelerate the progress towards universal health coverage in LMICs (45, 46). Lastly, bundled payment for rehabilitation services seems to be a new mechanism to reduce escalation of providers’ cost and increase productivity and health outcomes in the sector.

While many financing approaches we identified from HICs are focused on cost containment, a key challenge in LMICs is to expand the supply of rehabilitative services. Our review suggests that there are few studies that explore policy options to increase investment in the supply of services by promoting public-private partnership in the sector. Mechanisms of reimbursement are still based on global budget and salary which, like bundled payments, are helpful to control cost escalation but represent important barriers to expand supply and quality of services. Very few studies explore policies to increase the impact of rehabilitation services on individual productivity (38). There is ample room to combine financial incentives and non-financial benefits to expand labor supply and training. This suggests an opportunity for the introduction of technology to complement the existent labor supply and expand access to rehabilitation services. Our review also highlights the relevance of increasing educations grants and training grants to expand the supply of educational programs in rehabilitation.

Related to supply side challenges, our review points out fundamental challenges to weigh when designing an optimal package of health insurance coverage for rehabilitation services. Of critical importance is to balance the expansion of public health insurance coverage for rehabilitation services (47). The scope of the package to cover at the public and private sector is fundamental to control costs and should be done to include services that are context specific as well as show proven measured of cost-effective to the population. Reducing the possibility of adverse selection in the private and public insurance markets of individuals with disability is a difficult choice, and a top priority is to balance the responsibility between the private and public insurance system (48, 49). Lastly, designers of optimal packages of health insurance benefits must weigh the relevance of the inclusion of long-term services. All these factors involve difficult challenges and decisions. The current scholarly work does not provide clear answers to these issues. What it is important to highlight is the relevance of these elements when designing new mechanisms to fund rehabilitation services in LMICs.

An important consideration not previously discussed is the potential additional burden on health systems resulting from insufficient rehabilitation services. Limited or absent rehabilitation can lead to recurrent hospital admissions and the exacerbation of co-morbidities, significantly increasing overall healthcare costs. Moreover, there is an ethical dimension to this issue. While acute surgical and medical procedures are often financed, the subsequent rehabilitation, which is crucial for patient recovery and quality of life, may not receive equivalent financial support. This disparity raises ethical questions about the holistic care of patients and the allocation of healthcare resources. These aspects underline the need for a more integrated approach to healthcare financing, where rehabilitation is seen as an essential component of patient care, rather than an optional or secondary consideration.

Lastly, we found no studies that document subsidized or free rehabilitation services for the poor, and only one study on financial coping strategies due to high out-of-pocket cost of rehabilitation care (40). The study further attests the dire need for financial protection for people living with disabilities and effective financing mechanisms for rehabilitation services in LMICs. We also found no studies that explored public vs. private or public-private partnerships in financing rehabilitation services. However, a serial study in Sweden (36–39) reported a multi-sectoral partnership within the public sector in co-financing rehabilitation services. The study underlined that such collaboration might enhance the quality of services received by patients while also redesign the service delivery model. This review also highlights that the efficiency (estimated by mean cost per patient) of community/home-based rehabilitation compared to hospital/facility-based rehabilitation varies greatly among studies conducted in high income countries, while we did not find any studies comparing such programs in LMICs. Furthermore, financial incentives to promote faster recovery or increase productivity of private providers may be used to expand supply and quality of services (25).

This review is the first known review on financing models for rehabilitative care, inclusive of LMICs and HICs. A strength of our approach is the application of the PICO framework with the addition of “S” (supply). This framework served as a reference point to analyze included studies by policy-relevant questions.

Our review also has limitations. First, while our search strategy was comprehensive, the specific nature of our keywords and the focus on peer-reviewed literature might have limited our ability to capture all relevant studies, particularly those detailing specific financing models like microfinance and community finances by NGOs, as exemplified by the Aga Khan Foundation in Pakistan and the Rashtriya Swasthya Bima Yojana in India. Second, most included studies come from HICs, thus the applicability of the financing schemes outlined in this review to LMICs setting should be considered with caution. Finally, many of the included studies were published prior to 2010, which highlights the scarcity of evidence available and the urgency of building the evidence base to guide policy decisions.

Lastly, one notable limitation of this review is the exclusion of literature not published in English. This restriction may have resulted in missing significant contributions from studies conducted in lower and middle-income countries, where English is not the primary language of publication. This limitation could affect the comprehensiveness of our review and the generalizability of our findings to the LMIC context. Future reviews could address this gap by incorporating literature in multiple languages, thereby providing a more global perspective on the financing of rehabilitation services.

## Conclusion

This review reports various opportunities to expand rehabilitation services, namely through improvement of sustainable insurance mechanisms and packages, promotion of private investment and improvement in provider reimbursement mechanisms to bolster labor supply incentives as well expanding educational grants and the investment in public and private insurance schemes. It also identified that published literature highlighting financing models for rehabilitation services are extremely scarce, especially from LMICs. Most studies from HICs showed that user fees are still the main modality used in financing rehabilitation. This gap in knowledge should be addressed by subsequent research that can shed more light into the interventions, insurance mechanisms, and strategies to address challenges in rehabilitation financing. While optimal insurance package that is both context-specific and cost-effective is key in rehabilitation services, current literature provides limited specific examples of such models. This gap highlights a significant challenge for community-based rehabilitation professionals: balancing the diverse rehabilitation needs of various communities with the economic constraints of insurance packages.

## Data Availability

The original contributions presented in the study are included in the article/Supplementary Material, further inquiries can be directed to the corresponding author.
